# Safety of licensed vaccines in HIV-infected persons: a systematic review protocol

**DOI:** 10.1186/2046-4053-3-101

**Published:** 2014-09-11

**Authors:** Benjamin M Kagina, Charles S Wiysonge, Maia Lesosky, Shabir A Madhi, Gregory D Hussey

**Affiliations:** 1 Vaccines for Africa Initiative, Division of Medical Microbiology and Institute of Infectious Disease and Molecular Medicine, University of Cape Town, Cape Town 7700, South Africa; 2 Centre for Evidence-Based Health Care and Division of Community Health, Faculty of Medicine and Health Sciences, Stellenbosch University, Cape Town 7505, South Africa; 3 Department of Medicine, University of Cape Town, Cape Town 7925, South Africa; 4 Department of Science and Technology/National Research Foundation, Vaccine Preventable Diseases, University of the Witwatersrand, Johannesburg 2000, South Africa; 5 Medical Research Council: Respiratory and Meningeal Pathogens Research Unit, Faculty of Health Sciences, University of the Witwatersrand, Johannesburg 2193, South Africa; 6 National Institute for Communicable Diseases, National Health Laboratory Service, Centre for Vaccines and Immunology, 1 Modderfontein Road, Sandringham, Johannesburg 2131, South Africa

## Abstract

**Background:**

Safety of vaccines remains a cornerstone of building public trust on the use of these cost-effective and life-saving public health interventions. In some settings, particularly Sub-Saharan Africa, there is a high prevalence of HIV infection and a high burden of vaccine-preventable diseases. There is evidence suggesting that the immunity induced by some commonly used vaccines is not durable in HIV-infected persons, and therefore, repeated vaccination may be considered to ensure optimal vaccine-induced immunity in this population. However, some vaccines, particularly the live vaccines, may be unsafe in HIV-infected persons. There is lack of evidence on the safety profile of commonly used vaccines among HIV-infected persons. We are therefore conducting a systematic review to assess the safety profile of routine vaccines administered to HIV-infected persons.

**Methods/Design:**

We will select studies conducted in any setting where licensed and effective vaccines were administered to HIV-infected persons. We will search for eligible studies in PubMed, Web of Science, Cochrane Central Register of Controlled Trials (CENTRAL), Scopus, Africa-Wide, PDQ-Evidence and CINAHL as well as reference lists of relevant publications. We will screen search outputs, select studies and extract data in duplicate, resolving discrepancies by discussion and consensus.

**Discussion:**

Globally, immunisation is a major public health strategy to mitigate morbidity and mortality caused by various infectious disease-causing agents. In general, there are efforts to increase vaccination coverage worldwide, and for these efforts to be successful, safety of the vaccines is paramount, even among people living with HIV, who in some situations may require repeated vaccination. Results from this systematic review will be discussed in the context of the safety of routine vaccines among HIV-infected persons. From the safety perspective, we will also discuss whether repeat vaccination strategies may be feasible among HIV-infected persons.

**Systematic review registration:**

PROSPERO CRD42014009794.

## Background

Effective and safe vaccines against diseases such as severe forms of tuberculosis, diphtheria, tetanus, pertussis, mumps, measles, rubella, pneumococcus, polio, yellow fever, and rotavirus, among others, contribute towards preventing 2.5 million childhood deaths annually through vaccination [1]. Additionally, vaccines targeting adolescents (10–19 years old) such as human papillomavirus (HPV) vaccines, meningococcal conjugate vaccines, influenza vaccines as well as booster vaccines of measles, tetanus, diphtheria and pertussis are routinely used in some settings to mitigate vaccine-preventable diseases [2]. Therefore, public health strategies that target the vaccination of children, adolescents as well as adults are more likely to yield success in elimination of vaccine-preventable diseases as opposed to strategies that target children only [3].

The goal of any effective vaccine is to induce a long-lasting specific immunity that confers protection against the targeted pathogen. Some reports suggest that individuals with underlying HIV infection may have attenuated vaccine-induced immunity, including lower and loss of anamnestic responses, which could reduce the effectiveness of the vaccines [4]. Furthermore, a systematic review by Kerneis et al. showed that long-term immunity induced by many routinely used vaccines is diminished to non-protective levels in HIV-infected persons [5]. As a result, a repeat vaccination could be considered for certain vaccines in this population to ensure maintained protection against vaccine-preventable diseases. For example, the Advisory Committee on Immunization Practices (ACIP) recommended revaccination with measles, mumps and rubella (MMR) vaccine to HIV-infected persons over 12 months of age and with no evidence of immunosuppression [6].

The majority of HIV infections in infants and children occur early in life through vertical transmission from the mother, while in older age groups, HIV is acquired through horizontal transmission. HIV infection is prevalent in low-income and middle-income countries (LMICs) [7]. The LMICs account for about 85% of the global population and contribute to a disproportionately high burden of vaccine-preventable diseases (VPDs). Factors contributing to the high burden of the VPDs in LMICs include low rates of vaccine uptake [8,9], high rates of malnutrition [10], as well as high prevalence of underlying HIV infection; [7] all of which may result in lowering vaccine effectiveness in these settings.

The role of underlying immunosuppressive conditions contributing to reducing vaccine effectiveness is corroborated in some LMICs where a high burden of VPDs, despite reasonably high vaccination coverage, is reported [11]. Following the synthesised evidence by Kerneis et al., which showed that HIV infection results in diminished vaccine-induced immunity in the long term [5], our interest is to evaluate the feasibility of a repeat vaccination among HIV-infected persons from a safety perspective. We hypothesise that for some vaccines, HIV infection will compromise the safety profile and therefore, revaccination among children, adolescents and adults could be risky. This suggests that revaccination with some vaccines may not be feasible among HIV-infected persons. To test our hypothesis, we are conducting a systematic review. Our aim is to assess the safety profile of routinely used vaccines administered to HIV-infected persons.

The WHO recommends administration of most vaccines delivered through EPI to both HIV-uninfected and HIV-infected persons, with the exception of BCG where risk-benefit analysis needs to be considered. As far as we know, there is no systematic review on the safety profiles of many routinely used vaccines in HIV-infected persons.

### Primary objective

• The primary objective of this study is to compare the safety profile of the WHO-recommended vaccines administered to HIV-infected persons.

### Secondary objective

• The secondary aim is to compare the safety profile of the WHO-recommended vaccines re-administered to HIV-infected persons.

## Methods/Design

### Types of studies

We will consider only primary studies with the following designs:

• Interventional studies: individually randomised controlled trials (RCTs), cluster-randomised controlled trials (cRCT) and non-randomised control trials.

• Observational studies: case series, interrupted time series (ITS), controlled before-and-after (CBA) studies, cohort studies, case–control studies, cross-sectional studies and ecological studies.

Review articles, letters and editorials will be excluded.

### Study settings

We will include studies conducted in any setting and in any country, whether low-, middle- or high-income countries.

### Population

We will include children, adolescents and adults for this systematic review. Children will be defined as age category of 0–9 years. Adolescents will be defined as age category of 10–19 years, while adults will be defined as those above 19 years of age.

We will only include studies in which participants were HIV-infected or both HIV-infected and HIV-uninfected. Included studies must have used defined and standard assays or tests to determine the HIV infection. Studies that evaluated the safety of the vaccines prior to licensure will also be included provided the vaccines were licensed later.

### Types of interventions

Participants of studies included in this review must have received any WHO-recommended vaccine (Table 1).

**Table 1:** Summary of WHO position papers on the recommended vaccines

**Antigen**	**Children**	**Adolescents**	**Adults**
BCG			
Hepatitis B	3–4 doses	3 doses for high-risk groups if not previously vaccinated	
Polio	3–4 doses		
DTP	3 doses, booster (DTP), 1–6 years of age	Booster	Booster
*Haemophilus influenzae* type b (Hib)	3 doses with DTP		
Pneumococcal (conjugate)	3 doses with DTP		
Rotavirus	2 or 3 doses with DTP		
Measles	2 doses		
Rubella	1 dose	1 dose, adolescent girls if not previously vaccinated	
HPV	3 doses	Girls	
Japanese encephalitis	1 dose, booster dose after 1 year	Booster dose after every 3 years up to 10–15 years of age	
Yellow fever	1 dose		
Tick-borne encephalitis	3 doses		
Typhoid	1 dose or 3 doses (dependent on vaccine), booster dose after 3–7 years		
Cholera	3 doses with booster after every 6 months, 2 doses for children of 6 years and older/adults with booster every 2nd year		
Meningococcal	1 dose (1–29 years)		
Men A conjugate	2 doses (2–11 months) with booster 1 year after 1st dose		
Men C conjugate	2 doses (9–23 months), 1 dose (2 years and older)		
Quadrivalent conjugate			
Hepatitis B	At least 1 dose at 1 year or older		
Rabies	3 doses		
Mumps	2 doses		
Influenza (inactivated)	2 doses, revaccinate annually	1 dose from 9 years of age, revaccinate annually	

Vaccines for varicella virus, herpes zoster virus and pneumococcal (polysaccharides) are not in this table, but will be included in the review. This table is adapted from the Summary of WHO Position Papers—Recommendations for Routine Immunization. 1 ( http://www.who.int/immunization/policy/Immunization_routine_table1.pdf?ua=1).

The vaccines to be included in this review are as follows:

• Oral polio vaccine (OPV) and/or inactivated polio vaccine (IPV)

• Bacillus Calmette-Guérin (BCG)

• Diphtheria, pertussis and tetanus (DTP)—we will include both whole cell and acellular pertussis

• Rotavirus vaccine (RV)

• Hepatitis B vaccine (HBV)

• Hepatitis A vaccine (HAV)

• Measles vaccine or measles in combination with mumps and rubella (MMR) vaccine

• Yellow fever (YF)

• Pneumococcal conjugate vaccine (PCV)

• Pneumococcal polysaccharide vaccine

• Meningococcal vaccine

• Human papillomavirus vaccine (HPV)

• *Haemophilus influenzae* type b (Hib)

• Japanese encephalitis

• Tick-borne encephalitis

• Typhoid

• Cholera

• Rabies

• Influenza (inactivated)

• Varicella

• Herpes zoster virus vaccine

### Type of outcome measures

The primary outcome of this review is the establishment of the safety profile of licensed vaccines administered to HIV-infected individuals and the assessment of vaccine-related adverse events (frequency and duration of mild to severe adverse events).

### Search methods for identification of studies

A comprehensive search strategy has been developed for searching electronic and other resources. We will search the following electronic databases for primary studies: PubMed, Web of Science, Cochrane Central Register of Controlled Trials (CENTRAL), Scopus, Africa-Wide, PDQ-Evidence and CINAHL. The electronic search will use the common terms describing the safety of these routine vaccines. We will search for published articles with no language restriction. The detailed electronic search strategy for PubMed is provided in Table 2. In addition, we will search reference lists of relevant reviews and all potentially eligible studies.

**Table 2:** Proposed search strategy and search outputs for PubMed database

	**Query**
#5	(#3 AND #4)
#4	(HIV infected OR HIV positive OR HIV OR HIV exposed uninfected OR HEU)
#3	(#1 AND #2)
#2	(Persons OR Participants OR Newborns OR babies OR infants OR children OR adolescents OR teenage OR young adults OR youth OR adults)
#1	(Safety of OPV OR safety of IPV OR safety of polio vaccine OR effectiveness of OPV OR effectiveness of IPV OR effectiveness of polio vaccine OR efficacy of OPV OR efficacy of IPV OR efficacy of polio vaccine OR Safety of BCG vaccine OR safety of Bacillus Calmette Guérin vaccine OR effectiveness of BCG vaccine OR effectiveness of Bacillus Calmette Guérin vaccine OR efficacy of BCG vaccine OR efficacy Bacillus Calmette Guérin vaccine OR Safety of DTP vaccine OR safety of Diphtheria vaccine OR Safety of Pertussis vaccine OR safety of Tetanus vaccine OR effectiveness of DTP vaccine OR effectiveness of Diphtheria vaccine OR effectiveness of Pertussis vaccine OR effectiveness of Tetanus vaccine OR efficacy of DTP vaccine OR efficacy of Diphtheria vaccine OR efficacy of Pertussis vaccine OR efficacy of Tetanus vaccine OR Safety of RV vaccine OR safety of Rotavirus vaccine OR effectiveness of RV vaccine OR effectiveness of Rotavirus vaccine OR efficacy of RV vaccine OR efficacy of Rotavirus vaccine OR Safety of HBV OR safety of Hepatitis B vaccine OR effectiveness of HBV OR effectiveness of Hepatitis B vaccine OR efficacy of HBV OR efficacy of Hepatitis B vaccine OR Safety of HAV OR safety of Hepatitis A vaccine OR effectiveness of HAV OR effectiveness of Hepatitis A vaccine OR efficacy of HAV OR efficacy of Hepatitis A vaccine OR Safety of MMR vaccine OR safety of Measles vaccine OR safety of Mumps vaccine OR safety of Rubella vaccine OR effectiveness of MMR vaccine OR effectiveness of Measles vaccine OR effectiveness of Mumps vaccine OR effectiveness of Rubella vaccine OR efficacy of MMR vaccine OR efficacy of Measles vaccine OR efficacy of Mumps vaccine OR efficacy of Rubella vaccine OR Safety of YF vaccine OR safety of Yellow fever vaccine OR effectiveness of YF vaccine OR effectiveness of Yellow fever vaccine OR efficacy of YF vaccine OR efficacy of Yellow fever vaccine OR Safety of PCV OR safety of Pneumococcal conjugate vaccine OR effectiveness of PCV OR effectiveness of Pneumococcal conjugate vaccine OR efficacy of PCV OR efficacy of Pneumococcal conjugate vaccine OR Safety of MC vaccine OR safety of Meningococcal vaccine OR effectiveness of MC vaccine OR effectiveness of Meningococcal vaccine OR efficacy of MC vaccine OR efficacy of Meningococcal vaccine OR Safety of HPV vaccine OR safety of Human Papilloma Virus vaccine OR effectiveness of HPV vaccine OR effectiveness of Human Papilloma Virus vaccine OR efficacy of HPV vaccine OR efficacy of Human Papilloma Virus vaccine OR safety of JE vaccine OR safety of Japanese Encephalitis vaccine OR effectiveness of JE vaccine OR efficacy of JE vaccine OR safety of Hib vaccine OR safety of *Haemophilus influenzae* type b vaccine OR effectiveness of Hib vaccine OR efficacy of Hib vaccine OR safety of inactivated influenza vaccine OR effectiveness of inactivated influenza vaccine OR efficacy of inactivated influenza vaccine OR safety of cholera vaccine OR effectiveness of cholera vaccine OR efficacy of cholera vaccine OR safety of typhoid vaccine OR effectiveness of typhoid vaccine OR efficacy of typhoid vaccine OR safety of rabies vaccine OR effectiveness of rabies vaccine OR efficacy of rabies vaccine OR safety of Tick-Bone Encephalitis vaccine OR effectiveness of Tick-Bone Encephalitis vaccine OR efficacy of Tick-Bone Encephalitis vaccine OR safety of varicella vaccine OR effectiveness of varicella vaccine OR efficacy of varicella vaccine OR safety of herpes zoster virus vaccine OR effectiveness of herpes zoster virus vaccine OR efficacy of herpes zoster virus vaccine OR safety of pneumococcal polysaccharide vaccine OR effectiveness of pneumococcal polysaccharide vaccine OR efficacy of pneumococcal polysaccharide vaccine)

### Data collection and analysis

#### Selection of studies

Two authors will independently screen the search outputs for potentially eligible studies, compare their results and resolve disagreements by discussion and consensus. The two authors will then independently go through the full text of all potentially eligible studies to assess whether the studies meet the inclusion criteria as defined by the study design, setting, population, intervention and outcomes. Discrepancies in the list of eligible studies between the two authors will be resolved through discussion and consensus.

### Data extraction

A structured and standardised data collection form has been developed for extracting data from the selected studies. The form will capture key study characteristics, including methods, participants’ characteristics and outcomes (Additional file 1: Table S1). Prior to use, the extraction form will be piloted on at least four included studies identified randomly from the list of included studies, and if need be, modifications will be made. For the recently (2010 onwards) published literature, if any selected study has incomplete or missing data, we will contact the authors for more information. If the authors provide no additional information, a decision will be taken by at least two authors on the inclusion of the study in the final analyses.

### Assessment of risk of bias in included studies

We will use a similar approach to the one we previously described [12]. The quality of studies will be assessed using the Cochrane Collaboration’s tool for assessing risk of bias [13] for experimental studies and the Scottish Intercollegiate Guidelines Network (SIGN) checklist for other study designs [14].

### Grading the quality of evidence and strength of recommendations

We will use the Grading of Recommendations Assessment, Development and Evaluation (GRADE) system to assess the quality of evidence [15] (based on the clinical methods used to assess adverse events).

### Data synthesis

We will express the result of each study as a risk ratio (RR) with its corresponding 95% confidence intervals for dichotomous data or median difference (MD) with its standard deviation (SD) for continuous data. For each study, the adverse events in the vaccine arm will be compared with adverse events in the control arm with use of RRs and 95% confidence intervals (CIs). Pooled RRs and 95% CIs will be calculated for the effect of each vaccine on development of adverse events following immunisation using random effects model. Secondary analysis will compare the rates of adverse events following immunisation in HIV-infected versus HIV-uninfected with use of RRs and 95% CIs. Subgroup analyses will be conducted to investigate the rates of adverse events following revaccination relative to primary vaccination. We will conduct meta-analysis for studies that have used the same age group of participants, similar adverse events reporting method, same vaccines, time points post vaccinations and outcome measures where homogeneity of data allows. Heterogeneity will be assessed using the chi-squared test of homogeneity and quantified using the I-squared statistic (with 95% uncertainty intervals). Where possible, mixed effects models will be used to adjust for confounding factors such as co-morbidities and HIV-related protein and energy malnutrition (PEM).

### Assessment of heterogeneity

We anticipate substantial variation in study results due to differences in the study design, method of assessing adverse events, age group (children, adolescents and adults), study settings (low-income, middle-income, and high-income countries) and risk of bias. We will examine statistical heterogeneity between study results using the chi-squared test of homogeneity (with significance defined at the alpha level of 10%) and quantify any statistical heterogeneity between study results using the I-squared statistic [13].

### Assessment of reporting biases

A funnel plot will be used to investigate the risk of publication bias by vaccine, provided 10 or more studies are included in the analysis for each vaccine related adverse event evaluated. The funnel plot will be critically examined for asymmetry.

### Sensitivity analysis

We will conduct sensitivity analysis to establish if the meta-analysis results are influenced by clinical and methodological diversities. Subgroup analysis based on the CD4 cell counts and the viral load may be conducted if we have a sufficient number of studies reporting these variables.

## Discussion

We anticipate that our systematic review results will establish whether persons infected with HIV have an increased risk of adverse events following vaccination with routine vaccines. Our results may be used to guide vaccinologists in developing better vaccination strategies for HIV-infected populations. The results will be critical in the context of optimising current vaccination strategies in the context of HIV infection in any given setting [16].

## Competing interests

The authors declare that they have no competing interests.

## Authors’ contributions

BMK developed the study protocol and will conduct the initial search, screening of the search outputs, data extraction, data interpretation and manuscript preparation. CSW guided the development of the study protocol and will be consulted on studies that need to be included. ML wrote the data management and statistical analyses sections. SAM guided protocol development and will be consulted on the interpretation of the results and preparation of the manuscript. GDH conceived the study and guided protocol development; he will resolve disagreement on the selected studies for inclusion into the study and will be consulted with the interpretation of results and preparation of the manuscript. All authors read and approved the final manuscript.

## Supplementary Material

Additional file 1: Table S1 Proposed data extraction form. Click here for file
