# Supplementary material for: Safety of licensed vaccines in HIV-infected persons: a systematic review protocol
Source: Syst Rev. 2014 Sep 11;3:101. doi: 10.1186/2046-4053-3-101 (PMC4163552; doi:10.1186/2046-4053-3-101)
Supplement: Additional file 1: Table S1 — Proposed data extraction form. [file 2046-4053-3-101-S1.doc]

**Additional file 1Table S1: Proposed data extraction form**

## Details of person extracting the data

|  | Description | Comments |
| --- | --- | --- |
| Name of researcher completing the form |  |  |
| Date when form completed (dd/mm/yyyy) |  |  |

# *2) General information*

| Description of the study | Enter as appearing in the publication | Reference page/table or figure in the study |
| --- | --- | --- |
| Study ID based on surname of first author and year (e.g *Hussey 2001*) |  |  |
| Study DOI |  |  |
| Correspondence author and the contact details |  |  |
| Publication type | Full text  Abstract  Governmental or Book chapter non-governmental reports  Other (specify) |  |
| References of potentially eligible studies from the reference list | 1)  2)  3) |  |
| Notes/Comments |  | |

*3) Study eligibility assessment*

| Characteristics | Tick or describe as appropriate | Reference page/table or figure in article |
| --- | --- | --- |
| Primary study | Yes (Primary)  No |  |
| Type of study design |  |  |
| Study participants | Children  Adolescents  Adults  Other |  |
| Study setting | Low-income country  Lower middle-income country  Upper middle-income country  High-income country |  |
| Name of the country |  |  |
| Unit of allocation to the intervention  (*if applicable*) | Individual  Household  Cluster  Other (Specify) |  |
| Age of the participants at enrolment to the study (years) | Children (0-9)  Adolescents (10-19)  Adults (>19)  Multiple age groups |  |
| Informed consent obtained for study  (*if applicable*) | Yes No Unclear |  |
| Ethical approval obtained for study  (*if applicable*) | Yes No Unclear |  |
| Name of the vaccine/s used to immunize the study participants |  |  |
| Disease or diseases the vaccine/s is/are targeted for |  |  |
| Outcome measures | Yes  No  Frequency and/or duration of mild to severe adverse events after vaccination  Yes  No  Vaccine is currently licensed by WHO  Other (specify):  None |  |
| Final decision on study eligibility | Yes  No  (Include) (Exclude) |  |
| Reason(s) for exclusion |  | |

NB: Do not proceed to the next step if the study is excluded from the review

*4) Study aims and methods*

|  | Describe as stated in report/paper/book chapter | Reference page/table or figure in the study |
| --- | --- | --- |
| Aim(s) |  |  |
| Immunological assay used to test HIV infection |  |  |
| Age at which HIV test was done (years) |  |  |
| Definition of a positive HIV infection (If provided) |  |  |
| If HIV infected, CD4 counts performed? | Yes  No |  |
| Assay used to perform CD4 counts |  |  |
| Mean or median CD4 counts of the study group (specify mean or median) |  |  |
| Variance, IQR and range of CD4 counts (if provided) |  |  |
| If HIV infected, viral load performed? | Yes  No |  |
| Assay used to perform viral load |  |  |
| Mean or median viral load of the study group (specify mean or median) |  |  |
| Variance, IQR and range of viral load (if provided) |  |  |
| Vaccination date/day (*if multiple doses, different days, duplicate this row*) |  |  |
| Period between vaccine administration and safety assessment (*if multiple time points were evaluated, duplicate this row for each time point*) |  |  |
| Method used for safety evaluations if specified (*if different for multiple time points, duplicate this row and indicate the time points and assay used*) |  |  |
| Any intervention offered to the study participants | Yes No  Specify: |  |
| Notes: | | |

## 5) Participants

| Characteristics | Description | Reference page/table or figure in the study |
| --- | --- | --- |
| Study setting | Rural Urban Displaced community    Other (specify) |  |
| Socio-economic status of the participating populations | Low (L) Average (A) Above average (AA)    All (L, A &AA) Not clear |  |
| Methods used to classify the socio-economic status of the study population (if stated) |  |  |
| Participants HIV status at baseline | Infected Uninfected  (I) (UI) |  |
| Did HIV status change during the study period (for follow up studies)? | Yes  No |  |
| If Yes to above question, were the participants included in the final analysis? | Yes  No |  |
| Number of participants in each study group at baseline (if there are follow up time points, duplicate this row). Provide description of the group | n= |  |
| Age (mean in years) of the study participants at baseline |  |  |
| Birth weight (mean in Kgs)  (only for children studies) |  |  |
| Gender | Female Male Both |  |
| Ethnicity (if provided) |  |  |
| Notes (provide any other relevant information on the participants): | | |

*6) Outcome* measures

| Details of the outcome | Characteristics of the outcomes | | | | | Reference page/table or figure in the study |
| --- | --- | --- | --- | --- | --- | --- |
|  | Specific effectiveness of the vaccine: (indicate name of vaccine, e.g, measles) | | | | |  |
|  | Group  (if different vaccination strategies were used) | (proportion) % | (proportion) % | (proportion) % | Time point |  |
| Proportions of vaccinees developing the disease the vaccine was administered against (if stated)  (*NB: If incidence was evaluated for different vaccines or time points, duplicate these rows*. |  |  |  |  |  |  |
|  |  |  |  |  |  |  |
| Notes: | | | | | | |

| Details of the outcome | Characteristics of the outcomes | | | | | Reference page/table or figure in the study |
| --- | --- | --- | --- | --- | --- | --- |
|  | Mild and severe adverse events after vaccination with (indicate name of vaccine, e.g, measles) | | | | |
|  | Name of the adverse event: | Adverse events  (Females) | Adverse events  (Males) | Adverse events  (all in the group) | Time points |
| Frequency of adverse events reported  (*NB: If adverse events were evaluated for different vaccines or time points, duplicate these rows*. | 1)  2)  3) | Mild=  Severe= | Mild=  Severe= | Mild=  Severe= |  |  |
| Duration (in days) of adverse events reported  *NB: If adverse events were evaluated for different vaccines or time points, duplicate these rows*. | 1)  2)  3) | Mild=  Severe=  Other= | Mild=  Severe=  Other= | Mild=  Severe=  Other= |  |  |
| Notes | Specify other | | | | |  |

| Details of the outcome | Characteristics of the outcomes | | | Reference page/table or figure in the study |
| --- | --- | --- | --- | --- |
|  | Management/treatment of adverse events | | |  |
|  | Name of the adverse event: | Type of treatment (specify) | Time point |  |
| Number and proportions of vaccinees (n=) and (p=) | 1)  2)  3) |  |  |  |
|  |  |  |  |  |
| Notes: | | | | |

## 7) Risk of bias assessment

| Type of bias | Tick appropriately and describe below after the tick. | Reference page/table or figure in the study |
| --- | --- | --- |
| Is there selection bias? (Assess comparability of groups at baseline, confounding and adjustment. For RCTs assess sequence generation and allocation concealment). | Yes No Unclear |  |
|  |
| Is there performance bias? (Assess fidelity of the interventions, and quality of the information regarding who received which interventions, including blinding of study subjects and healthcare providers) | Yes No Unclear |  |
|  |
| Is there detection bias? (Assess whether there was biased and correct appraisal of outcomes, including blinding of assessors) | Yes No Unclear |  |
|  |
| Is there attrition bias? (Assess the completeness of sample, follow-up and outcome data, reasons for loss to follow up explained) | Yes No Unclear |  |
|  |
| Is there reporting bias (Assess selective reporting of results) | Yes No Unclear |  |
|  |
| Other biases (specify) | Yes No Unclear |  |
|  |

## 8) Other relevant information

|  | Descriptions/figures as stated in report/paper/book chapter | Reference page/table or figure in the study |
| --- | --- | --- |
| Key conclusions from the authors |  |  |
| Notes |  |  |
